# Effects of Soil Salinity on Sucrose Metabolism in Cotton Fiber

**DOI:** 10.1371/journal.pone.0156398

**Published:** 2016-05-26

**Authors:** Jun Peng, Lei Zhang, Jingran Liu, Junyu Luo, Xinhua Zhao, Helin Dong, Yan Ma, Ning Sui, Zhiguo Zhou, Yali Meng

**Affiliations:** 1 Key Laboratory of Crop Physiology & Ecology in Southern China, Ministry of Agriculture, Nanjing Agricultural University, Nanjing, China; 2 State Key Laboratory of Cotton Biology, Institute of Cotton Research, Chinese Academy of Agricultural Sciences, Anyang, China; 3 Zhejiang Chinese Medical University, Hangzhou, China; National Key Laboratory of Crop Genetic Improvement, CHINA

## Abstract

Cotton (*Gosspium hirsutum* L.) is classified as a salt tolerant crop. However, its yield and fiber quality are negatively affected by soil salinity. Studies on the enzymatic differences in sucrose metabolism under different soil salinity levels are lacking. Therefore, field experiments, using two cotton cultivars, CCRI-79 (salt-tolerant) and Simian 3 (salt-sensitive), were conducted in 2013 and 2014 at three different salinity levels (1.15 dS m^-1^ [low soil salinity], 6.00 dS m^-1^ [medium soil salinity], and 11.46 dS m^-1^ [high soil salinity]). The objective was to elucidate the effects of soil salinity on sucrose content and the activity of key enzymes that are related to sucrose metabolism in cotton fiber. Results showed that as the soil salinity increased, cellulose content, sucrose content, and sucrose transformation rate declined; the decreases in cellulose content and sucrose transformation rate caused by the increase in soil salinity were more in Simian 3 than those in CCRI-79. With increase in soil salinity, activities of sucrose metabolism enzymes sucrose phophate synthase (SPS), acidic invertase, and alkaline invertase were decreased, whereas sucrose synthase (SuSy) activity increased. However, the changes displayed in the SuSy and SPS activities in response to increase in soil salinity were different and the differences were large between the two cotton cultivars. These results illustrated that suppressed cellulose synthesis and sucrose metabolism under high soil salinity were mainly due to the change in SPS, SuSy, and invertase activities, and the difference in cellulose synthesis and sucrose metabolism in fiber for the two cotton cultivars in response to soil salinity was determined mainly by both SuSy and SPS activities.

## Introduction

Salinity is one of the major environmental problems that challenges the plant growth and development in both developed and developing countries [1]. Cotton (*Gossypium hirsutum* L.) is an important natural fiber and oil crop. It, also, provides edible protein for livestock feed. Although cotton is classified as a salt-tolerant crops, with a salinity threshold of 7.7 dS m^-1^, the fiber strength, micronaire value, maturity ratio, and maturity percentage are drastically reduced with increase in salinity[2, 3]. Cotton is considered the most important textile crop grown worldwide[4]. Therefore, fiber yield and quality are the main criteria for cotton production.

Each cotton fiber is a single cell, which initiates at or just before the anthesis, in the ovule epidermis. Following the initiation, single-celled lint fibers rapidly elongate to 2.5–3.0 cm for about 16 d, after which they switch to intensive secondary cell wall cellulose synthesis in the tetraploid species [5]. By the time they reach maturity, more than 90% of the fiber dry weight is cellulose [6]. Cotton fiber formation is primarily a cellulose synthesis process, which requires many enzymes and organic molecules [7, 8]. Sucrose synthase (SuSy, E.C. 2.4.1.13) can both synthesize and degrade sucrose, but its function in fiber is primarily degrading sucrose to provide UDP-glucose as the substrate for cellulose synthesis [9]. Moreover, SuSy is an anaerobic protein and can provide additional sugar during hypoxia or anaerobic conditions [10]. In addition, acidic/alkaline invertases (E.C. 3.2.1.26) can catalyze the hydrolysis of sucrose, providing carbon and energy for cellulose synthesis [11]. Acid invertase (AI), especially, catalyzes the irreversible hydrolysis of sucrose to glucose and fructose. Sucrose phosphate synthase (SPS, E.C. 2.4.1.14), which is considered as the key enzyme affecting cellulose synthesis, regulated sucrose synthesis [12, 13]. A part of fructose, released in the process of sucrose degradation by SuSy to provide UDP-glucose for cellulose synthesis, may be recycled to sucrose through SPS [14].

Thus, cellulose, sucrose, and β-1,3-glucan are closely associated for ascertaining main fiber quality attributes. Extensive investigations have been conducted on the enzymology of sucrose metabolism in marine planctomycete *Rhodopirellula baltica* [15] and Methylotrophic bacteria [16], under salinity stress. However, there are no previous reports on the relationship between the metabolism of substances involved in fiber development and the fiber quality attributes under salt stress. Therefore, the aim of the present study was to examine the effect of soil salinity on dynamic changes in the contents of fiber development-related substances (cellulose and sucrose), as well as to analyze their relationships with the fiber quality attributes (length and strength). This study aimed to reveal the physiological mechanism of the effect of soil salinity on the fiber quality attributes from the perspective of the metabolism of fiber development-related substances and intended to provide a theoretical basis for the improvement of fiber quality and breeding of ecologically stable cultivars.

## Materials and Methods

### Experimental design

The field experiments were carried out using two cotton cultivars that were significantly different in their salt tolerance. CCRI-79, a salt-tolerant cultivar and Simian 3, a salt-sensitive cultivar [17] were grown in Dafeng Basic Seed Farm located in Dafeng (33°20′N, 120°46′E), Jiangsu in 2013 and 2014. This farm is state-owned and no specific permissions were required for using this farm for the experiments conducted in the present study. Moreover, the field studies did not involve experiments with endangered or protected species. Three soil types, similar in texture and nutrient composition (Table 1), but having low, medium, or high salinity were selected. Their conductivities were 1.15 (low soil-salinity, LS), 6.00 (medium soil-salinity, MS), and 11.46 dS m^-1^ (high soil-salinity, HS). The LS and MS soils had been planted with rice for the previous 5 and 2 years, respectively in contrast to the HS soil, which was not planted with rice or cotton before the experiment. Cotton seeds were directly sown in the field at a density of 45,000 hm^-2^ on April 28, 2013 and May 4, 2014. Three replicates were assigned randomly for each treatment. Nitrogen (N)-fertilizer (240 kg N ha^-1^) was subsequently added to the field; 40% was added as the base fertilizer and 60% during flowering and boll formation. The amount of P used as the base fertilizer was 120 kg P_2_O_5_ ha^-1^. The amount of K-fertilizer was 120 kg K_2_O ha^-1^, which was used at a ratio of 50% as the base fertilizer and 50% during flowering and boll formation.

**Table 1:** Physical and chemical properties of the soils that were tested, in years 2013 and 2014.

Index		2013			2014	
	LS	MS	HS	LS	MS	HS
TN (g·kg^-1^)	1.11	1.07	1.12	1.13	1.08	1.10
AN (mg·kg^-1^)	98.92	98.15	99.25	97.58	97.25	98.42
AP (mg·kg^-1^)	27.83	28.94	27.05	27.35	27.86	26.75
AK (mg·kg^-1^)	122.73	121.55	122.12	123.01	122.14	122.86
BD (g·cm^-3^)	1.23	1.22	1.23	1.22	1.23	1.22
EC (dS·m^-1^)	1.15	6.00	11.46	1.15	6.00	11.46

TN: Total nitrogen; AN: Available nitrogen; AP: Available phosphorus; AK: Available potassium; BD: Bulk density, EC: Electrical conductivity, LS: low soil salinity, MS: medium soil salinity, HS: high soil salinity.

### Measurements and methods

White flowers at sympodial fruiting branches at main-stem nodes 6–7 of the cotton plants were tagged with small plastic tags listing the flowering date. About 6–8 cotton bolls with the same anthesis date of each treatment were picked at 17, 24, 31, and 38 days post-anthesis (DPA) in 2013 and 2014. Boll wall, seeds, and fiber of the sampled boll were separated and cotton fiber were immediately placed in liquid nitrogen and stored at −80°C for further analysis.

### Cellulose and sucrose content analyses

The fiber was digested with an acetic acid-nitric acid mixture, and the cellulose content was measured by anthrone method according to Updegraff [18].Sucrose from the cotton fiber was extracted and quantified according to the method of Pettigrew [19]. Fiber sample (0.3g dry weight, DW) was extracted thrice with 5 ml of 80% ethanol. The ethanol extracts were incubated in an 80°C water bath for 30 min, centrifuged at 10,000 ×*g* for 10min, and three aliquots of the supernatant were collected and pooled together for sucrose measurements [14]. The sucrose assay was conducted by the method of Hendrix [20].

### Enzymatic analyses

Enzyme extraction and assay for SuSy, invertases and SPS activity were performed according to the method of King et al. [21].

### Measurements of fiber quality

Tagged bolls were hand harvested after bolls opened and ginned, and arranged in individual groups according to fruiting branches. Ginned fiber from each group was sent to the Cotton Quality Supervision, Inspection, and Testing Center of China Ministry of agriculture for quality analysis. Fiber quality including fiber upper-half mean length (UHML), uniformity index (UI), strength and micronaire of each lint sample were determined by a high volume instrument at the Cotton Quality Supervision, Inspection, and Testing Center of China Ministry of Agriculture.

### Statistical analysis

OriginPro 9.0 was used for the data processing and forming figures. The data were subjected to an ANOVA using SPSS version 17.0. The data are presented as the means ± standard errors of at least three independent experiments, and the different letters indicate the statistical differences at P < 0.05. The coefficient of variation (CV) was calculated as the ratio of the standard deviation (including all treatments) to mean.

## Results

### Quality of cotton fiber

A number of the fiber quality traits including fiber upper-half mean length (UHML), fiber strength (ST), and micronaire value (MIC) were affected considerably by soil salinity in both 2013 and 2014 (Table 2). With the increase in soil salinity UHML, MIC, and ST decreased in both CCRI-79 and Simian 3. However, elongation percentage (EL) and uniformity index (UI) were not affected by soil salinity in any of the two years. When data from both the years were combined and the fiber quality was analyzed, there were significant variation in UHML and UI according to the year, cultivars, salinity, and the interactions among them, such as years × cultivars, years × salinity, cultivar × salinity, and years × cultivars × salinity. In addition, there were highly significant differences (P < 0.01) in all the examined fiber quality traits between the cultivars and among different salinity levels, except no significant difference in EI between the cultivars. The two cultivars, CCRI-79 and Simian 3, had different ranges of UHML, ST, and MIC in different soil salinity. The CVs of UHML, ST, and MIC for Simian 3 were greater than those of CCRI-79. These results indicated that cotton fiber quality was more affected by soil salinity in Simian 3 than in CCRI-79.

**Table 2:** Effects of soil salinity on fiber quality of the two cotton cultivars in years 2013 and 2014.

Year	Cultivars	Salinity levels	UHML ^a^	UI ^b^	MIC ^c^	EL ^d^	ST ^e^
			(mm)	(%)			(cN tex^-1^)
2013	CCRI-79	LS	27.8a	82.3a	5.5a	6.7a	27.2a
		MS	27.2b	82.1a	5.3b	6.8a	26.4b
		HS	26.2c	82.0a	4.5c	6.6a	25.8c
		CV(%) ^f^	2.98	0.18	10.38	1.49	2.65
	Simian 3	LS	27.6a	82.1a	5.4a	6.7a	28.1a
		MS	26.4b	82.0a	5.0b	6.8a	27.0b
		HS	24.5c	81.8a	4.2c	6.8a	25.1c
		CV(%)	5.97	0.19	12.56	0.85	5.68
2014	CCRI-79	LS	27.5a	83.3a	5.4a	6.7a	26.9a
		MS	26.9b	83.1a	5.1b	6.9a	26.4b
		HS	26.1c	82.9a	4.8c	6.8a	26.0c
		CV(%)	2.62	0.24	5.88	1.47	1.71
	Simian 3	LS	27.3a	83.1a	5.4a	6.8a	27.8a
		MS	26.0b	82.9a	4.9b	6.9a	26.8b
		HS	25.2c	82.3a	4.4c	6.7a	25.6c
		CV(%)	4.05	0.5	10.2	1.47	4.12
		Year (Y)	**	**	**	NS	NS
		Cultivar (C)	**	**	**	*	**
		Salinity (S)	**	**	**	**	**
		Y*l	*	*	*	NS	NS
		YSl	*	**	NS	NS	**
		C*l	**	**	**	NS	**
		Y*lin	**	**	NS	**	**

^a^ UHML, fiber upper-half mean length;

^b^ UI, uniformity index;

^c^ MIC, micronaire value;

^d^ EL, elongation percentage;

^e^ ST, fiber strength;

^f^ CV, coefficient of variation.

* and ** indicate significant differences at P#0.05 and 0.01 probability levels, respectively. ns, not significant (P≤0.05). Values followed by a different letter between salinity levels are significantly different at P = 0.05 probability level. Each value represents the mean of three replications. LS: low soil-salinity, MS: medium soil-salinity, and HS: high soil-salinity.

### Cellulose and sucrose content in cotton fiber

**Fig 1:**
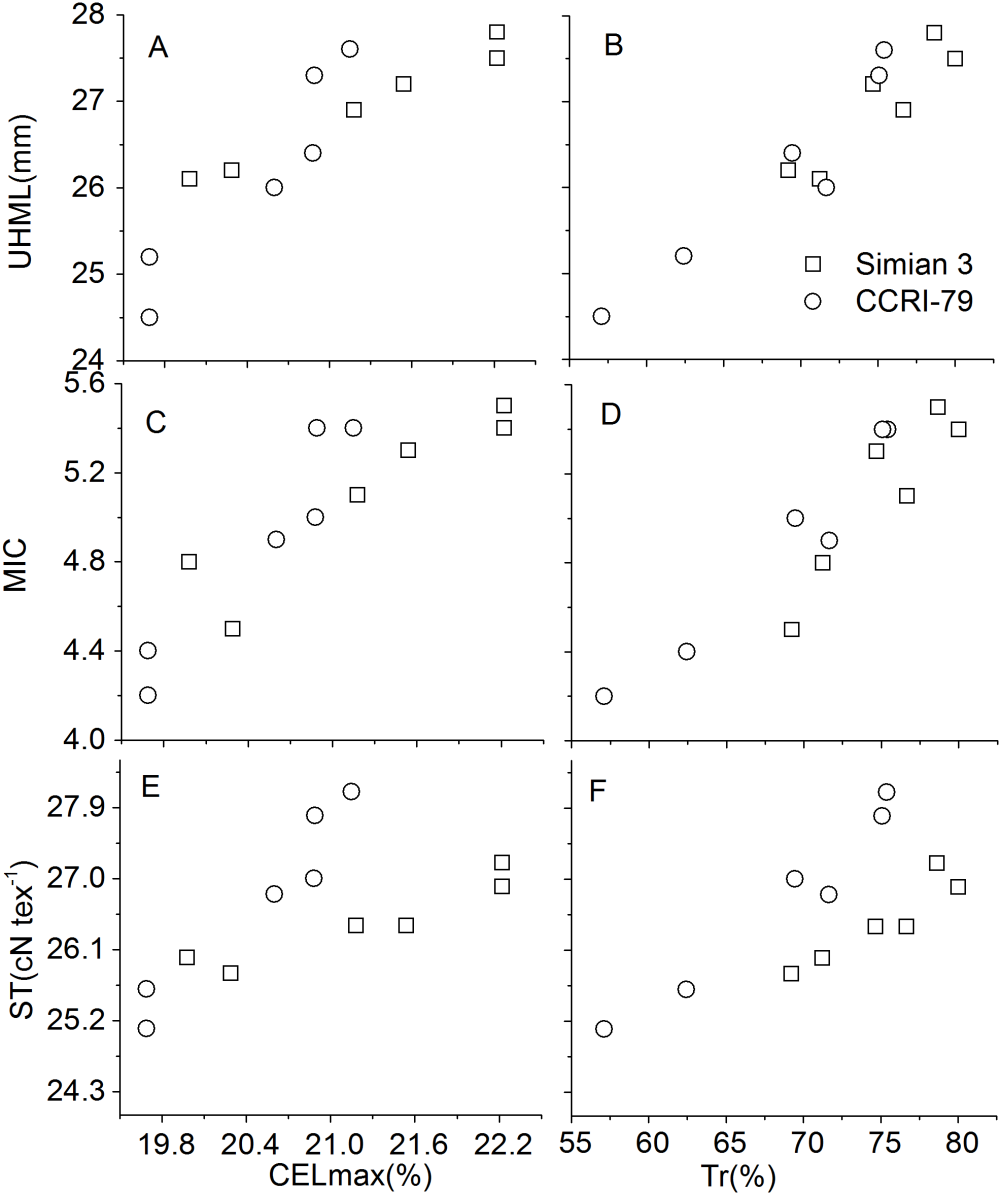
Correlation coefficients among parameters in fibers of two cotton cultivars during 2013–2014. UHML-fiber upper-half mean length, MIC-micronaire value, ST-fiber strength, CELmax-maximum cellulose content, Tr-sucrose transformation rate. * and **, significant differences at P = 0.01 and P = 0.05 probability levels, respectively. n = 6, R_0.05_ = 0.811, R_0.01_ = 0.917.

Fibers cellulose content increased from 17 DPA onwards as shown in Table 3. The content varied under different soil salinity conditions. In both the cultivars, CCRI-79 and Simian 3, the cellulose content under high soil salinity (HS) was lower than that under medium soil salinity (MS) and low soil salinity (LS). In the fibers of 38 DPA, the fiber cellulose content under HS conditions was 14%–21% and 17%–42% lower than that under MS and LS conditions, respectively. The coefficient for the correlations between fiber length and maximum cellulose content, fiber strength and maximum cellulose content, and fiber micronaire values and maximum cellulose content were 0.989**,0.936** and 0.927** (**P<0.01), respectively, in CCRI-79, and 0.938**, 0.964** and 0.957**(**P<0.01), respectively in Simian 3 (Fig 1A, 1C and 1E).

**Table 3:** Effects of soil salinity on cellulose content, sucrose content, and sucrose transformation rate in cotton fibers of the two cotton cultivars, in years 2013 and 2014.

Year	Cultivars	Salinity levels	Cellulose content (%)				Sucrose content (mg g^-1^ DW)				Tr
			17DPA	24DPA	31DPA	38DPA	17DPA	24DPA	31DPA	38DPA	%
2013	CCRI-79	LS	25.28 a	45.37 a	66.53 a	75.42 a	16.15 a	10.29 a	4.33 a	3.45 a	78.65 a
		MS	21.20 b	42.75 b	61.90 b	71.26 b	13.25 b	9.85 b	4.22 a	3.35 a	74.68 b
		HS	19.88 c	38.56 c	55.32 c	62.00 c	10.92 c	7.76 c	3.75 b	3.31 a	69.21 c
		CV%	12.73	8.13	9.20	9.88	19.5	14.53	7.51	2.14	6.38
	Simian 3	LS	23.80 a	44.36 a	61.17 a	65.56 a	13.71 a	7.23 a	4.42 a	3.37 a	75.40 a
		MS	18.78 b	38.78 b	54.62 b	57.90 b	10.83 b	5.72 b	4.08 a	3.29 a	69.46 b
		HS	17.86 c	34.83 c	48.04 c	49.59 c	7.38 c	4.85 b	2.03 b	3.20 a	57.11 c
		CV%	15.87	12.18	12.02	10.93	29.81	20.28	36.72	2.68	13.86
2014	CCRI-79	LS	23.47 a	52.80 a	73.97 a	80.07 a	18.86 a	10.13 a	8.93 a	3.77 a	80.01 a
		MS	22.11 a	45.37 b	66.53 b	75.42 b	16.43 b	6.98 b	6.39 b	3.73 a	76.67 b
		HS	20.64 b	39.10 c	58.75 c	62.94 c	12.81 c	5.87 c	5.73 b	3.69 a	71.23 c
		CV%	6.41	14.99	11.46	12.17	18.99	28.86	24.08	1.11	5.83
	Simian 3	LS	22.62 a	48.72 a	70.33 a	77.25 a	14.91 a	8.49 a	6.95 a	3.71 a	75.09 a
		MS	21.97 a	43.25 b	61.17 b	65.56 b	13.01 b	6.09 b	4.16 b	3.68 a	71.66 b
		HS	17.82 b	27.91 c	50.34 c	54.15 c	9.77 c	5.02 b	4.06 b	3.67 a	62.43 c
		CV%	12.5	26.99	16.51	17.59	20.68	27.22	32.44	0.62	9.39

Note: Tr, sucrose transformation rate; DPA, days post anthesis; Values followed by a different letter between salinity levels are significantly different at P = 0.05 probability level. Each value represents the mean of three replications. LS: low soil-salinity, MS: medium soil-salinity, and HS: high soil-salinity.

Fiber sucrose content decreased from 17DPA onwards (Table 3). The content under HS conditions was lower than that under MS and LS conditions. The sucrose contents between HS, MS, and LS on 17 DPA were significant different for Simian3 and CCRI-79 during 2013 and 2014. The reduction in sucrose content on 38DPA among the cultivars grown under different soil salinity conditions was not significantly different. This finding illustrated that the sensitivity of sucrose content to soil salinity was significantly affected at the beginning of cellulose synthesis. Moreover, the coefficients for the correlation between fiber length and sucrose transformation rate, fiber strength and sucrose transformation rate, and fiber micronaire values and sucrose transformation rate were 0.910*, 0.931** and 0.924** (**P<0.01, *P<0.05), respectively, in CCRI-79, and 0.958**, 0.975**and 0.975** (**P<0.01), respectively in Simian 3. This indicted that sucrose transformation rate was positively correlated with fiber quality (Fig 1B, 1D and 1F).

With increasing soil salinity, the sucrose transformation rate showed a decreasing trend and was consistent between the years (Table 3). Under soil salinity stress, the content of sucrose and cellulose as well as the transformation of sucrose declined. However, the extent of change in the two cultivars under different salinity conditions was different. In Simian 3, the cellulose content ranged from 17.86%to 65.56% in 2013 and from 20.10%to 77.25% in 2014.The sucrose transformation rate in this cultivar ranged from 57.11%to 75.40% in 2013 and from 62.43% to 75.09% in 2014. The CV for the sucrose transformation rate in Simian 3 under different soil salinity conditions were more than that in CCRI-79. This indicated that Simian 3 was more sensitive to soil salinity than CCRI-79 with respect to the cellulose synthesis and sucrose metabolism in the cotton fibers.

### Activities of enzymes of sucrose metabolism

**Fig 2:**
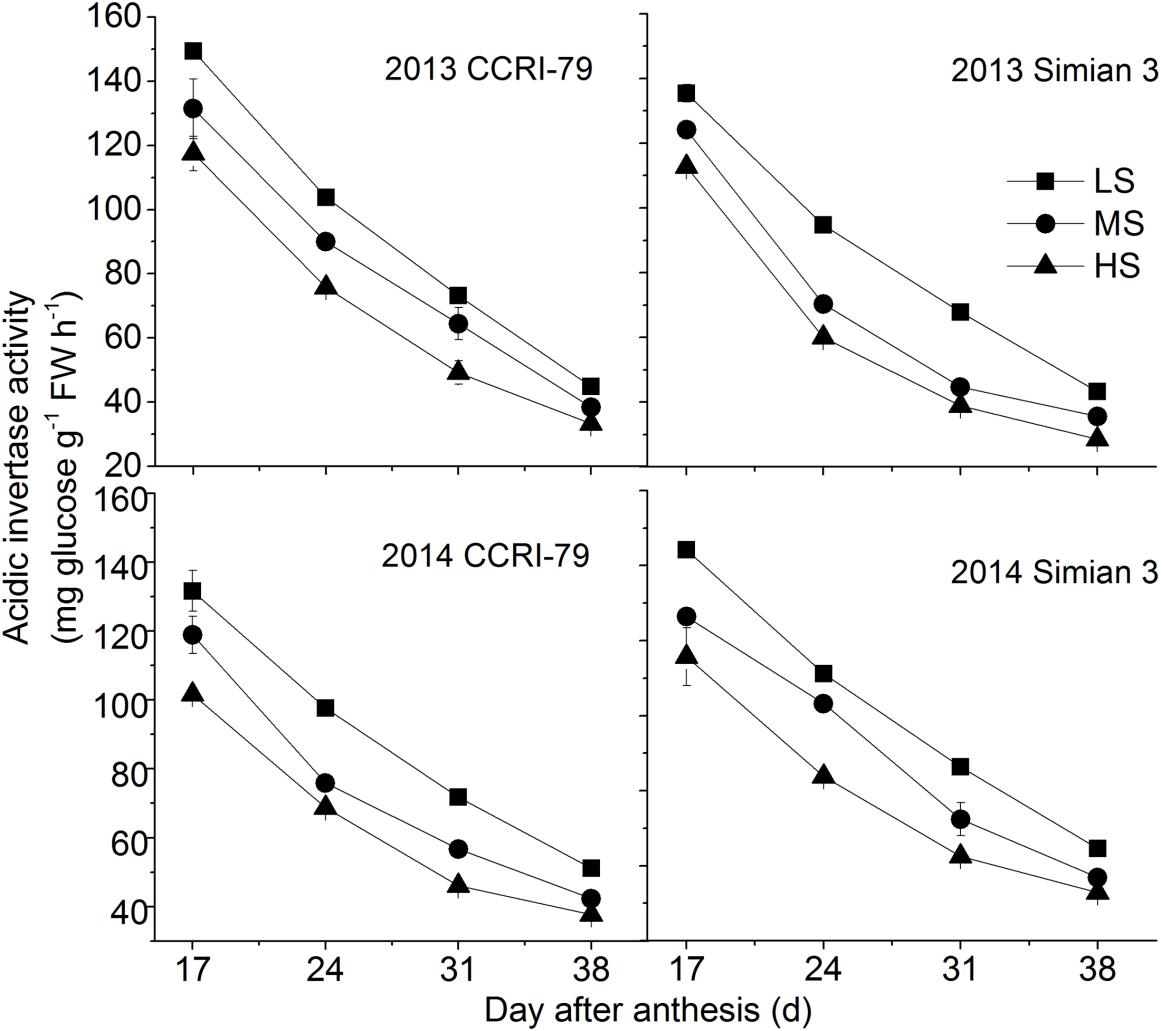
Effects of soil salinity on dynamics of acidic invertase activity in cotton fiber with cultivars of CCRI-79 and Simian 3 in 2013 and 2014. LS: low soil-salinity, MS: medium soil-salinity, and HS: high soil-salinity. Vertical bars represent ± standard error (n = 3).

**Fig 3:**
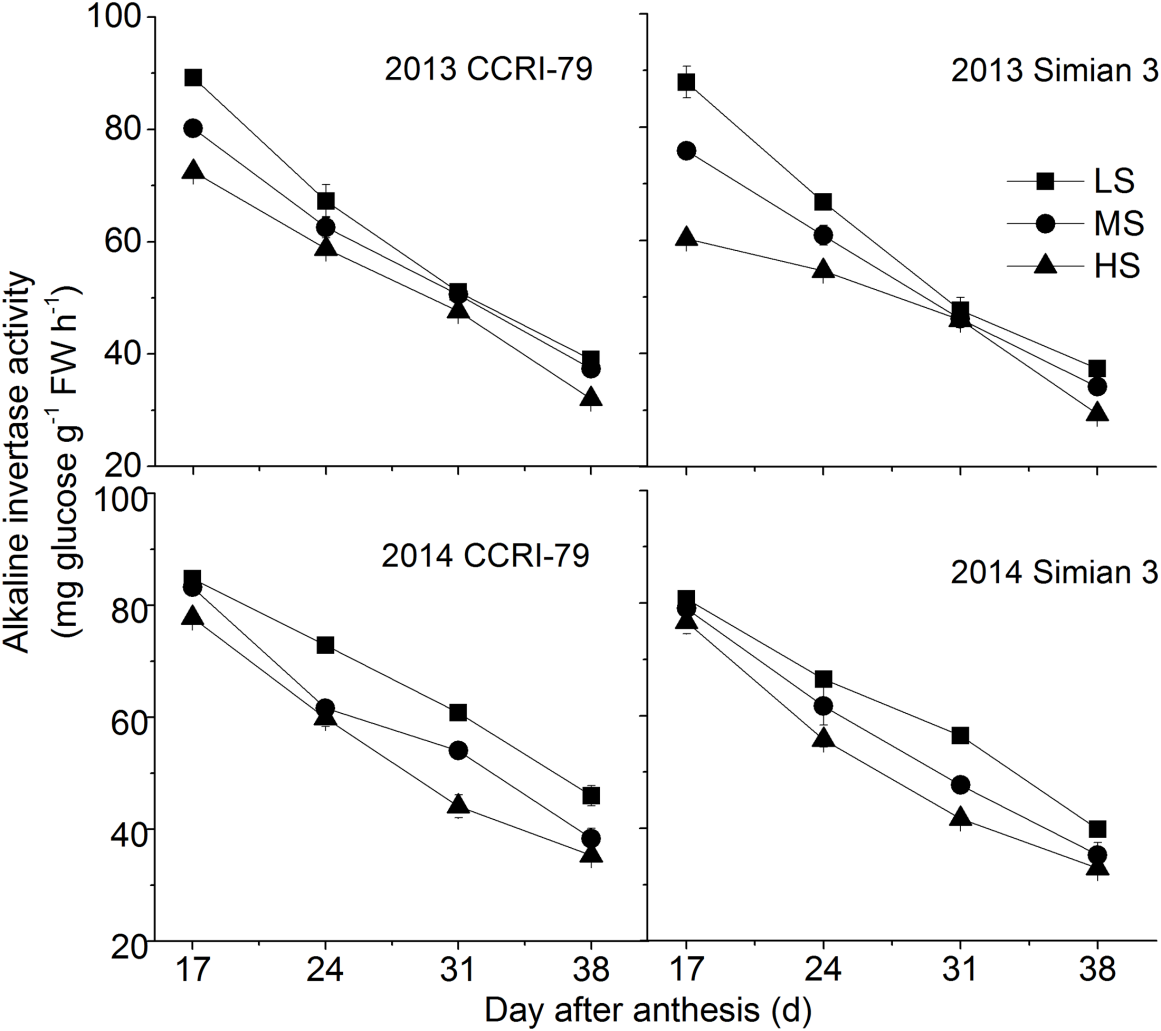
Effects of soil salinity on dynamics of alkaline invertase activity in cotton fiber with cultivars of CCRI-79 and Simian 3 in 2013 and 2014. LS: low soil-salinity, MS: medium soil-salinity, and HS: high soil-salinity. Vertical bars represent ± standard error (n = 3).

The acid invertase activity in cotton fibers was higher than that of alkaline invertase. However, change in acid and alkaline invertase activities were similar and showed a decrease with the fiber development. (Figs 2 and 3). Furthermore, a significant decrease in the activity of both the enzymes was observed with increase in soil salinity.

**Fig 4:**
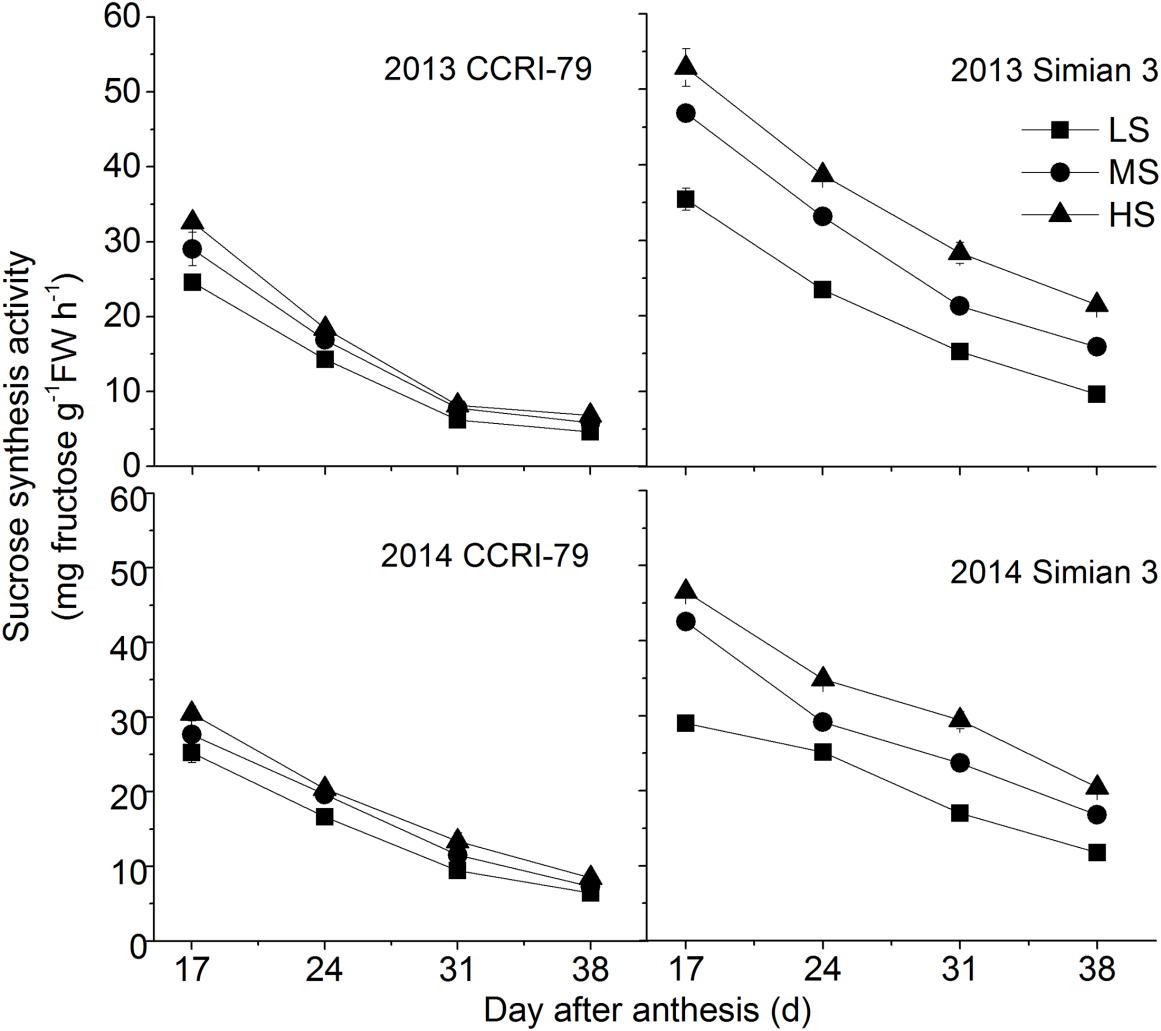
Effects of soil salinity on dynamics of sucrose synthesis activity in cotton fiber with cultivars of CCRI-79 and Simian 3 in 2013 and 2014. LS: low soil-salinity, MS: medium soil-salinity, and HS: high soil-salinity. Vertical bars represent ± standard error (n = 3).

Salinity enhanced SuSy activity of the fibers; the changes in response to salinity were influenced by the developmental age of cotton fibers (Fig 4). The SuSy activity in Simian 3 was higher than that in the CCRI-79 cultivar. It was observed that soil salinity caused a general increase in the activity of sucrose degrading enzyme; the response of this enzyme to soil salinity was same for both the cultivars.

**Fig 5:**
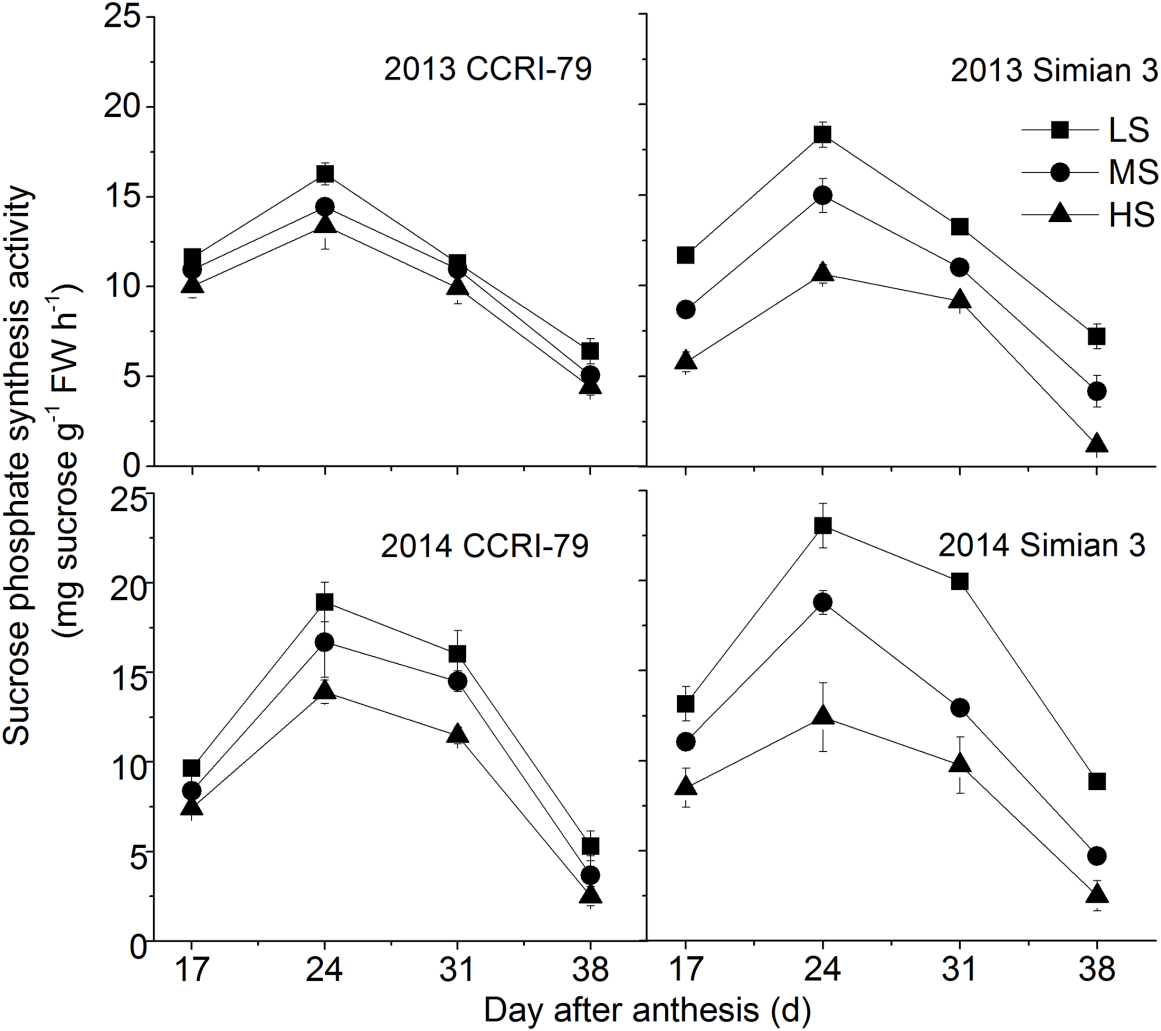
Effects of soil salinity on dynamics of sucrose phosphate synthase activity in cotton fiber with cultivars of CCRI-79 and Simian 3 in 2013 and 2014. LS: low soil-salinity, MS: medium soil-salinity, and HS: high soil-salinity. Vertical bars represent ± standard error (n = 3).

As shown in Fig 5, the activity of SPS increased after 17 DPA until it peaked, and then declined with the fiber development. The peak values for the SPS activity differed in the two cultivars under the different soil salinity conditions. In Simian 3, the SPS activity reduced with the increased salinity whereas no differences were observed in the SPS activities under different soil salinity conditions in CCRI-79.

An analysis of the CV% of the activities of the enzymes that are involved in sucrose metabolism (such as SuSy, SPS, and acid/alkaline invertase) revealed a differential effect of soil salinity on sucrose metabolism enzymes. Among the four sucrose metabolizing enzymes, SuSy and SPS were most significantly affected by soil salinity whereas no differences were observed in the acid/alkaline invertase activities under different soil salinity conditions (Table 4).

**Table 4:** Comparisons of coefficients of variations on the activities of sucrose metabolism enzymes in cotton fibers of the two cultivars at different soil salinity levels, in years 2013 and 2014.

Year	DPA(d)	Acid invertase activity		Alkaline invertase activity		SuSy activity		SPS activity	
		CCRI-79	Simian 3	CCRI-79	Simian 3	CCRI-79	Simian 3	CCRI-79	Simian 3
2013	17	12.07	15.08	10.42	10.42	13.99	19.67	7.35	33.79
	24	15.60	19.05	6.77	6.77	12.53	24.25	9.91	26.35
	31	19.48	19.05	3.78	3.78	13.75	30.31	6.66	18.48
	38	14.84	18.21	10.06	10.06	19.62	37.78	19.01	71.51
2014	17	12.81	13.22	4.47	2.61	9.37	23.36	13.25	21.40
	24	18.64	17.78	10.91	8.83	10.51	16.46	15.19	29.61
	31	22.23	22.25	15.90	15.31	17.00	26.50	16.54	36.72
	38	15.52	15.56	13.82	9.77	13.54	26.58	36.69	60.08

DPA, day post anthesis.

## Discussion

Previous studies have documented that fiber length is largely dependent on genetic factors, whereas fiber maturity properties, which depend on photosynthesis in the fiber cell wall, are more sensitive to changes in the growth environment [22]. However, several studies have indicated that the fiber quality is influenced by soil salinity [23]. Thus, fiber quality such as length, strength, and micronaire etc. are probably affected by the changing growth environment. However, while assessing the effects of soil salinity on the fiber characteristics of cotton cultivars that differed in their salt tolerance, Ashraf and Ahmad (2000) found that as the soil salinity increased, the fiber fineness increased, but the fiber maturity and fiber strength decreased [24]. In addition, the salt-tolerant cultivars had higher fiber maturity and fiber strength than the salt-sensitive cultivars [25]. Therefore, in order to understand the difference in fiber formation between the salt-tolerant and salt-sensitive cultivars under different levels of soil salinity, we performed field experiments in 2013 and 2014. Our results showed that fiber length, strength and micronaire values decreased as the soil salinity increased, which were consistent with those reported by Ashraf and Ahmad [24].

More than 85% of the constituents of a mature cotton fiber is cellulose, the synthesis and accumulation of which mainly occurs during secondary wall thickening of the fibers, and is closely related to the fiber strength [26].Secondary wall deposition begins towards the end of fiber elongation as a result of enhanced cellulose synthesis; callose synthesis in the fibers reaches a peak during this period [27]. In the present study, the correlation coefficient of cellulose content with the key fiber properties indicated that fiber length, was positively correlated with increase in cellulose content, and so did fiber strength and micronaire. This suggests that high fiber quality is correlated with the maximum cellulose content and the correlation is influenced by the effects of soil salinity.

Soil salinity has been demonstrated to significantly affect the strength and length of cotton fibers; these attributes of the fiber are also directly regulated by sucrose metabolism [28, 29]. SuSy and invertase are both involved in sucrose cleavage in sink tissues, and can be considered as biochemical markers of the sink strength. The results of present revealed that the activities of fiber acid and alkaline invertase were restricted by soil salinity, whereas fiber sucrose synthase activities were highest under HS. Increased sucrose cleavage by SuSy in response to stress conditions contributed to the production of more ATP from the same amount of sucrose, which was consistent with the report of Chao et al. [30].

The reduced transformation rate of sucrose under saline conditions was the result of fortified residual sucrose content compared to the available sucrose content. This illustrated that there was adequate sucrose in cotton fibers under saline conditions but it could not be effectively utilized for cellulose synthesis. Sucrose is degraded to provide UDP-glucose (UDPG) for cellulose synthesis catalyzed by SuSy, which is a critical component in the high-rate of cellulose synthesis in secondary-walls [31]. Sucrose metabolism, a key process for cellulose synthesis, is sensitive to soil salinity in many plants [32, 33]. Pettigrew (2001) found that sucrose level in cotton fibers was low and reduced under unfavorable environmental conditions when compared to other soluble carbohydrates[34]. In the present study, when cotton plants were subjected to high soil salinity, the sucrose content and sucrose transformation rate in the fibers decreased; these results were in agreement with those reported in the above-mentioned study. In addition, the coefficients for the correlation between sucrose transformation rates and the key fiber properties were highly correlated in the present study, which indicated that soil salinity affect the sucrose transformation process form sucrose to cellulose to weaken the fiber qualities.

Cotton cultivars have different sensitivity levels to soil salinity [35]. The change in the contents of cellulose and sucrose and the sucrose transformation rate in response to soil salinity differed between the CCRI-79 and Simian 3 cultivars in the present study. CCRI-79 was salinity-tolerant, whereas Simian 3 was partially sensitive to salinity stress. These results indicated that different cultivars employ different physiological strategies against salinity stress. The ability to accumulate high amounts of cellulose and enhancement of the sucrose transformation rate might be important for fiber development under salinity stress.

Sucrose metabolism involves many enzymes that contribute to cellulose synthesis in cotton fibers. Our results demonstrate that SPS, SuSy, and acid/alkaline invertase activities, which are involved in sucrose metabolism were restrained by soil salinity. This could be a probable reason for the reduction in sucrose transformation and cellulose content in cotton fibers under salinity stress.

SuSy is a critical enzyme in cellulose synthesis at high rates in the secondary walls. In both CCRI-79 and Simian 3, SuSy activities under HS were significantly higher than that under LS. M-SuSy is especially important for high rate of cellulose synthesis in secondary walls [36]. More than 70% suppression of SuSy activity in the ovule epidermis was reported to be the cause of a fibreless phenotype and it is suggested that the changes in SuSy activity could be related to the increase or decrease in cellulose synthesis [4]. However, in the present study, we found that the high level of SuSy activity in HS did not improve the cellulose synthesis. This might be attributed to the existence of two types of SuSy in cotton fibers; a proportion of M-SuSy converts into S-SuSy under salt stress conditions. The observed increase in SuSy activity in the present study could be mostly because of the increased S-SuSy activity, which supplies UDPG for general metabolic needs, but does not have a vital role in the partitioning of carbon to cellulose [37]. Sucrose might be degraded to produce free UDPG by S-SuSy activity, but it could not be used in the cellulose synthesis [14]. However, no differences was observed between the two cultivars in terms of change in the SuSy activity, which indicated that this was not the reason for the observed difference in cellulose synthesis and sucrose metabolism in cotton fibers under soil salinity conditions.

A previous study demonstrated that a high invertase activity was required for cotton fiber elongation mediated through the osmotic pathways. Fibers with higher invertase activity had faster elongation rates [38]. In *Arabidopsis*, the increased sucrose contents in the sink due to the over-expression of sucrose phosphate synthase gene was ascribed to the elongation of internodes, greater stem diameters, and longer fibers compared to wild-type plants [39]. Acid invertase, the usual isoform of invertase in the cell walls and vacuoles, might be the major player in the plant’s response to stress conditions [40]. In the present study, with the increase in soil salinity, the invertase activity decreased, indicating that this soil salinity-mediated decrease in the invertase activity influences the source-to-sink discharging of sucrose, eventually weakening the sink strength.

SPS, an enzyme involved in the sucrose synthesis in cotton fibers, is very sensitive to soil salinity and its activity is influenced by the biotic and abiotic stress [14]. Haigler et al. (2007) found that over-production of SPS in cotton increased the sucrose synthesis and improved the fiber quality under controlled environmental conditions [14, 27]. In the present study, under soil salinity stress, SPS activity in the fibers decreased. As evident from Table 4, SPS was the most sensitive enzyme among the four sucrose metabolizing enzymes (SuSy, acid invertase, alkaline invertase, and SPS) under salinity stress. The SPS activity in the two cotton cultivars had different sensitivity to soil salinity; the Simian 3 SPS being more sensitive. Due to the decrease in SPS activity, flux from fructose to sucrose decrease, which leads to an increase in fructose content in the fibers and suppression in M-SuSy activity; this results in as adverse effect on the cellulose synthesis [41]. This was positively related to the difference in cellulose content and the sucrose transformation rate in fibers of the two cultivars under salinity stress. We assumed that the decline in the SPS activity in cotton fibers under soil salinity would impede the flux of sucrose from glucose, thereby reducing the synthesis of sucrose and cellulose.

Furthermore, as shown in Table 4, we discovered that the CV% for the differences in the SuSy and SPS activities were higher than those for the activity of acid and alkaline invertase. These results indicate that the SuSy and SPS activities, which are part of sucrose metabolism, were easily affected by soil salinity. In addition, the SuSy and SPS activities in the two cotton cultivars had different sensitivities to soil salinity, with higher CV% observed in Simian 3 than in CCRI-79, indicating that the Simian 3 enzymes are more sensitive to salinity. These observations indicate that the differences in the activities of SuSy and SPS in the two cotton cultivars under soil salinity might be the reason for their differential sensitivities to salinity stress with regard to cellulose synthesis and sucrose metabolism.

Several reports indicate that field management strategies such as nitrogen, potassium fertilization, mulching, and irrigation [42, 43] could compensate for the potential yield loss and increase the tolerance of plants against soil salinity. Therefore, elucidation of the mechanisms of tolerance against different levels of soil salinity and methods for improving the fiber quality under salinity stress are urgently needed. In the present study, we observed that soil salinity reduced the cellulose synthesis and sucrose utilization by down-regulating the activities of the key sucrose metabolizing enzymes. Moreover, examination of the effect of soil salinity on fiber development in the two cotton cultivars showed that Simian 3 was more sensitive to soil salinity than CCRI-79.

## Conclusions

With the increase in soil salinity, fiber length, fiber strength, and micronaire values decreased. Highly correlated coefficients between these critical fiber quality and the cellulose content and sucrose transformation rates revealed a close relationship between them, under the effect of soil salinity. Soil salinity hampered cellulose synthesis, decreased the sucrose transformation rate, and influenced the activities of sucrose metabolizing enzymes in the two cotton cultivars examined in the present study. Differences in the sucrose metabolism and cellulose synthesis were observed in the two cultivars. As CCRI-79 was more salt-tolerant than Simian 3, the cellulose and sucrose content, sucrose transformation rate, and activities of SuSy and SPS changed slightly with increased soil salinity in this cultivar. The present study indicates that sucrose metabolism ascertains the cellulose synthesis under saline conditions in cotton fibers, with SuSy and SPS being the critical enzymes for synthesis. An understanding of the effects of soil salinity on cotton fiber development will aid in the breeding of new salt tolerant cotton cultivars.
